# Biocompatible Carbon-Based Coating as Potential Endovascular Material for Stent Surface

**DOI:** 10.1155/2018/2758347

**Published:** 2018-10-04

**Authors:** Magdalena Wawrzyńska, Iwona Bil-Lula, Anna Krzywonos-Zawadzka, Jacek Arkowski, Mikołaj Łukaszewicz, Dariusz Hreniak, Wiesław Stręk, Grzegorz Sawicki, Mieczysław Woźniak, Marek Drab, Kaja Frączkowska, Maciej Duda, Marta Kopaczyńska, Halina Podbielska, Dariusz Biały

**Affiliations:** ^1^Department of Emergency Medical Service, Faculty of Health Sciences, Wroclaw Medical University, Wroclaw, Poland; ^2^Department of Medical Laboratory Diagnostics, Division of Clinical Chemistry, Wroclaw Medical University, Wroclaw, Poland; ^3^Institute of Low Temperature and Structure Research, Polish Academy of Sciences, Wroclaw, Poland; ^4^Department of Pharmacology, College of Medicine, University of Saskatchewan, Saskatoon, SK, Canada; ^5^USI, Unit of Nanostructural Bio-Interactions, Hirszfeld Institute of Immunology and Experimental Therapy, Polish Academy of Sciences, Wroclaw, Poland; ^6^Department of Biomedical Engineering, Faculty of Fundamental Problems of Technology, Wroclaw University of Science and Technology, Wroclaw, Poland; ^7^Department and Clinic of Cardiology, Faculty of Postgraduate medical Training, Wroclaw Medical University, Wroclaw, Poland

## Abstract

Stainless steel 316L is a material commonly used in cardiovascular medicine. Despite the various methods applied in stent production, the rates of in-stent restenosis and thrombosis remain high. In this study graphene was used to coat the surface of 316L substrate for enhanced bio- and hemocompatibility of the substrate. The presence of graphene layers applied to the substrate was investigated using cutting-edge imaging technology: energy-filtered low-voltage FE-SEM approach, scanning electron microscopy (SEM), Raman spectroscopy, and atomic force microscopy (AFM). The potential of G-316L surface to influence endothelial cells phenotype and endothelial-to-mesenchymal transition (EndoMT) has been determined. Our results show that the bio- and hemocompatible properties of graphene coatings along with known radial force of 316L make G-316L a promising candidate for intracoronary implants.

## 1. Introduction

Percutaneous coronary intervention (PCI), based on metallic stent implantation, is now widely utilized for the treatment of atherosclerotic lesions and myocardial infarction (MI) [1]. Intracoronary stent placement allows for the resolution of lumen stenosis/obstruction of the epicardial artery and restoration of optimal blood flow. Stainless steel is commonly used for stent production due to its excellent mechanical properties, such as its radial force. 316L is one of the materials commonly used for balloon expandable stent type, the golden standard for PCI [2]. Nevertheless, as a consequence of metallic nature of the material, the interaction of living cells with the implant surface can lead to a strong immunological response, such as proliferation of smooth muscle cells [3] and fibrocytes [4]. This results in atheromatous neointimal layer formation, renarrowing of the vessel lumen (restenosis), and impairment of the implant function [5]. In-stent restenosis (ISR) in coronary arteries remains an unresolved issue of the long-term outcome of PCI procedures. Bare metal stents (BMS) were originally associated with a high frequency of restenosis, which affected about 20-30% of patients at 6 months after stent implantation [6]. The next generation, drug eluting stent (DES), was developed to locally deliver cytotoxic or cytostatic drugs in order to reduce smooth muscle cell proliferation and to inhibit the inflammatory process [7]. Although the introduction of DES greatly reduced the incidence of ISR, it still remains a major complication after stent implantation. Despite the improvements in stent placement techniques and product design enhancements, the incidence of ISR among patients with second-generation sirolimus-eluting stents is 10.6% [8]. Endothelial cells play a crucial role in mitigation of the vascular response to stent implantation [9]. Moreover, endothelial-to-mesenchymal transition (EndoMT) due to inflammation-stimulated production of TGF-*β* has recently emerged as another possible source of tissue “myofibroblasts”. EndoMT is a biological process in which endothelial cells lose their specific phenotype markers such as vascular endothelial cadherin and acquire a mesenchymal or myofibroblastic phenotype. They also express mesenchymal cell products such as *α* smooth muscle actin (*α*-SMA) and type I collagen [10, 11] leading to undesirable fibrosis in vascular wall. Besides acquisition of an activated profibrogenic phenotype, these cells are capable of migrating into surrounding tissues [9]. Moreover, the lack of functioning endothelial layer covering the stent struts facilitates the direct contact of blood cells with the implant surface. The blood-exposed bare metal stent may activate the platelet-derived clot formation and acute in-stent thrombosis [12].

To mitigate the adverse physiological response to implanted material, surface modification of the biomedical implant is necessary to enhance its bio- and hemobiocompatibility. Graphene is a single atomic sheet of conjugated sp2 carbon atoms with unique physical, mechanical, and chemical properties. Recently, a great potential of graphene and its derivatives, such as graphene oxide (GO), for medical applications has also been raised [13]. Graphene has been used in many biomedical applications, including the culture of neuronal cells and human osteoblasts [14, 15]. It has effectively been demonstrated that molecular coating of graphene on clinical grade nitinol is more bio- and hemocompatible than uncoated nitinol [16].

In this investigation a PMMA-based transfer method is used to obtain graphene layers on a medical-grade 316L stainless steel surface. The process, developed primarily for silicon target substrates, was optimized towards improvement of graphene adhesion and transfer rate to the stainless steel surface. The presence of successfully applied graphene layers on the steel alloy substrate was confirmed using a set of cutting-edge imaging techniques, by energy-filtered low-voltage FE-SEM approach [17], scanning electron microscopy (SEM), Raman spectroscopy, and atomic force microscopy (AFM).

We showed that graphene coated stainless steel (G–316L) meets the functional criteria of caridiovascular implant surface by enhancing endothelial cell growth leading to better cell proliferation. Moreover, we have demonstrated the potential of the graphene layer to control the pathophysiology of endothelial cells by preventing the transformation of endothelial cells into fibrocyte-phenotype cells. This feature supports the mechanisms of proper endothelial layer formation on the implant surface. We also showed a decreased activation of blood platelets in contact with G-316L compared to 316L. The hemo- and biocompatible properties of graphene layer, along with excellent mechanical properties of 316L, may give the G-316L implants a promising future in the production of cardiovascular stents.

## 2. Materials and Methods

### 2.1. Preparation and Characterization of 316L Steel Discs Covered with Graphene Layer

For sample preparation, surgical stainless steel (316L) circular discs with a diameter of 10 mm were polished mechanically to obtain a flat surface for the transferred graphene layer. In order to improve adhesion, the surface of the steel substrate has been subjected to hydrophilization in 30% hydrogen peroxide hot bath. The high-quality CVD-grown monolayer graphene on Cu substrate used in the experiment was supplied by NanoCarbon, Warsaw. The graphene monolayer has been moved to the substrate using the PMMA-based method, including drop-coating of the PMMA solution in trichloromethane on the graphene side of the Cu foil, etching the copper layer with iron (III) nitrate solution, carefully placing the Cu-PMMA stack onto the target substrate and removal of the polymer layer with hot acetone vapor. For the improvement of the graphene layer adhesion, two annealing steps were introduced. Before the removal of the PMMA, the steel-G-PMMA stack was baked at 200°C for 1 hour; after the acetone etching, the steel with graphene on top was further annealed in 90°C for 5 hours.

### 2.2. Raman Spectroscopy

The Raman measurements were performed under 488 nm laser line in backscattering geometry using a RenishawInVia Raman microscope equipped with a confocal DM2500 Leica optical microscope and a CCD detector.

### 2.3. Scanning Electron Microscopy (SEM)

Preliminary SEM images for evaluation of successful graphene transfer procedure (Figures 2(a) and 2(b)) were taken using a FEI NovaNanoSEM 230 FE-SEM microscope at 10kV (Figure 2(a)) and 5kV (Figure 2(b)) of accelerating voltage.

### 2.4. Low-Voltage Scanning Electron Microscopy Studies (Low-Voltage SEM)

Scanning electron microscopy of steel or graphene-overlaid discs was processed at low accelerating voltage (800 V) of the primary beam without any coating of the samples, as described by Hodyra-Stefaniak et al. [17]. The samples were washed in water and dehydrated in series of methanol (25%-50%-75%-100%-100%) in one-hour steps at 4°C. Samples underwent critical point drying with methanol exchanged for liquid CO_2_ in an automatized approach, (CPD300 AUTO, Leica Microsystems, Austria) and imaged with a cross-beam scanning electron microscope equipped with Schottky field-emission cathode (Auriga 60, Carl Zeiss, Oberkochen, Germany) at 0.8 kV accelerating voltage; thus, the imaging was performed within a mode referred to as the low-voltage, field-emission scanning electron microscopy (LV-FE-SEM) of nonlabeled, critical point-dried sample. Dual-imaging mode was applied with simultaneous recording of secondary electrons SE2 (Everhart-Thornley detector), SE1 (In-Lens detector), and backscattered electrons energy-filtered with low cut-off electrostatic grid filter (energy-selective BSE detector, EsB), set at 760 V. The EsB detector ensured the electron yield as a function of the retarding (negative) cut-off potential of the detector grid. The retarding potential was set to a level of energy 40 eV lower than the energy of the primary beam (760 V retarding potential filter when incident beam at 800 V), depending on the sample. For all imaged samples the material contrast was achieved with EsB detector by recording the backscattered electrons with low energy losses (low-loss BSE, LL-BSE) in real time, simultaneously with other detectors. The material contrast (EsB with retarding grid potential) could be reliably correlated with topographic features (ET and In-Lens detectors) on the samples and served as the major source of information about the graphene coating or biological objects on studied substrate.

### 2.5. Atomic Force Microscopy (AFM)

The surfaces covered with graphene were imaged by atomic force microscopy (AFM) using a Multi-Mode IIId scanning probe microscope with the extender module. Height measurements of the objects were performed with an instrumental vertical resolution of ±0.1 nm. Commercially available silicon cantilevers (Bruker AFM Probes, Camarillo, CA, USA) were used for tapping mode scanning with typical resonance frequency in the range of 183–192 kHz and a spring constant of 48 N/m. The set-point amplitude of the cantilever was maintained by the feedback circuitry to 90 % of the free oscillation amplitude of the cantilever. The scan angle was maintained at 0°, and the images were captured in the trace direction with a scan rate between 1.000 and 1.600 Hz. All samples were measured at room temperature in air. Data analysis was performed after flattening and plane-fit auto, height measurements based on the cross-sectional profiles, and/or analysis.

### 2.6. Endothelial Cell Culture and Treatment Conditions

The human primary coronary artery endothelial cells (HPCAEC) obtained from the American Type Culture Collection (ATCC; Rockville, MD) were cultured in Vascular Cell Basal Medium containing Endothelial Cell Growth Kit-BBE (0,2% (v/v) Bovine Brain Extract (BBE), 2% (v/v) fetal bovine serum, 5 ng/mL rh EGF, 10 mM L-glutamine, and 0.75 Units/mL heparin sulfate), supplemented with 10 *μ*g/mL gentamicin and 25 *μ*g/mL amphotericin B solution (Sigma Aldrich). The cell line was cultured at 37°C in a water-saturated 5% CO_2_ atmosphere. Cells were passaged at confluence and used within the first five passages.

The steel (316L) and the graphene-overlaid (G-316L) discs (*ϕ*=10 mm) used in all experiments were decontaminated with 70% ETOH, washed 3 times with PBS and placed on a 24-well culture plates (Grainer Bio-One, Cellstar). Then the HCAEC at density of 5×10^4^ cells/cm^2^ were seeded and cultured for 24h or 72h.

### 2.7. Endothelial Cells Adhesion Assay on Graphene Coated 316L (G-316L)

To determine the adhesive function of graphene coating, after 24h of culturing, the cells were washed 3 times with PBS, fixed with 4% (w/v) paraformaldehyde (15 min), and permeabilized with PBS containing 0.1% (v/v) Triton X-100, 1% (w/v) BSA, 10% (v/v) normal goat serum, and 0.3M glycine for one hour in RT. Then the cells were incubated with the mouse monoclonal anti-endothelial cell antibody [BW-200] (Abcam, ab15605, 1*μ*g/ml in 1% (w/v) BSA in PBS) overnight at 4°C and with goat anti-mouse IgG H&L antibody (DyLight 550, Abcam) at 1/500 dilution in PBS containing 0,2 % (w/v) BSA for 45 min at RT, in a dark and humid chamber. DAPI at concentration of 1.43 *μ*M (Sigma Aldrich) was used to stain the cell nuclei. Eclipse E600 fluorescence microscope (Niko, Minato-ku, Japan) was used to count the number of cells.

### 2.8. Assessment of Endothelial Cells Proliferation on Graphene Coated 316L (G-316L)

To assess the proliferation of HCAEC after 24h and 72h, the cells were fixed with ice cold 10% (w/v) trichloroacetic acid for 1h in 4°C, washed (5 times) with ddH_2_O, air dried and stained with 250 *μ*l 4% (w/v) sulforhodamine B solution in 1% acetic acid for 30 min. The unincorporated dye was removed with 1% acetic acid while the incorporated dye was then liberated from the cells with 10 nM Tris base solution. After gentle stirring, 200 *μ*l of solution was transferred into 96-well plate and the absorbance was measured spectrometrically at 490 nm. The absorbance was proportional to total biomass, corresponding to cell proliferation rate.

### 2.9. Evaluation of Cytotoxicity of Graphene Coated 316L (G-316L) and Cell Viability Test

To assess the cytotoxicity of graphene coated discs, the commercial In Vitro Toxicology Assay Kit from Sigma Aldrich was used. The test was performed according to the manufacturer's instruction.

To discriminate viable and nonviable HPCAEC, the trypan blue staining of cells after 24h and 72h of cultivation was performed. Cell viability was calculated as the number of viable cells divided by the total number of cells within the grids on the hemocytometer.

### 2.10. Assessment of Metabolic Activity of Endothelial Cells Cultivated on Graphene Coated (G-316L) 316L Substrate

To determine the number of viable, metabolically active cells, the simultaneous double-staining procedure, using fluorescein diacetate (FDA, Sigma Aldrich) and DAPI, was used. Briefly, the HCAEC were cultured on 316Land G-316L discs for 72h (5x10^4^ cells/cm^2^), washed 3x with PBS, and stained with 1 *μ*M FDA for 15 min, 37°C in a dark and humid chamber. DAPI was used to stain the cell nuclei. Nikon Eclipse E600 fluorescence microscope was used to estimate the bright green fluorescent viable cells. The measured signal served as indicator of cell metabolic activity, as the conversion of FDA is esterase dependent.

### 2.11. Immunohistochemistry and Image Analysis

Briefly, cells were seeded at density of 1×10^4^ cells/cm^2^ on 316L and G-316L discs and cultured for 24h and 72h under conditions described above. The cells were fixed in 4% (w/v) paraformaldehyde in PBS pH 7.4, permeabilized with PBS containing 0.1% (v/v) Triton X-100, and incubated with mix of mouse monoclonal anti-endothelial cell antibody [BW-200] (Abcam, ab15605, 1ug/ml in 1% (w/v) BSA in PBS) and rabbit polyclonal anti-FAP-*α* (Abcam, ab28244, 10 *μ*g/ml in 1% (w/v) BSA in PBS) overnight at 4°C. The discs were washed and stained with secondary antibody: goat anti-mouse IgG H&L antibody (DyLight 550, Abcam) and donkey anti-rabbit IgG H&L antibody (DyLight 488, Abcam), respectively, at 1/500 dilution in PBS containing 0,2 % (w/v) BSA for 45 min at RT, in a dark and humid chamber. DAPI was used to stain the cell nuclei. After washing, the discs were transferred on a glass slide and mounted with Fluoromount Aqueous Mounting Media (Sigma Aldrich). The cells were counted with Nikon Eclipse E600 fluorescence microscope, captured with a video camera module (Canon, EOS 650D), and analyzed with ImageJ 1.50b14. Briefly, color images were first converted to 8-bit image in grey scale (IrfanView, version 4.40). Then, the area of positive immunostaining was estimated by the number of RGB pixels within measuring mode, by separate measurements with red, green, and blue channels of RGB image. Thus, the area of positive reaction was determined as the percentage of red, green, and blue pixels area.

### 2.12. Hemocompatibility Assessment of Graphene Coated Surface

The hemocompatibility of G-316L in comparison to 316L was evaluated by the assessment of platelet adhesion and activation. Blood samples for platelets preparation were obtained from a volunteer, who declared lack of any diseases and anticoagulants administration. Blood was collected by venipuncture to tube containing 3.8% (wt/vol.) sodium citrate (9:1) and immediately centrifuged at 100 x g for 20 min (37°C) to obtain platelet-rich plasma (PRP). 2/3 of PRP fraction was transferred to a new 15 ml tube and centrifuged at 100 x g for 20 min (37°C) to pellet of contaminating red and white blood cells. The number of platelets in PRP was assessed by use of Cell-Dyn 1800 (Abbott); 500 *μ*l of PRP containing 2.0 x 10^5^ of platelets was transferred onto each plate. Platelets were allowed to adhere to graphene covered (n=6) and steel discs (n=3) for 15 min at 37°C. Nonadherent platelets were removed by rinsing the discs with PBS (3 times). Subsequently, adherent platelets were fixed with 10% PFA (10 min at RT), rerinsed three times with PBS, permeabilized with 0.1% Triton X-100 for 5 min at RT, washed again, and stained with conjugated phalloidin-FITC (5 *μ*g/ml) for 20 min. After staining, platelets were washed three times with PBS and photographed by Nikon Eclipse E600. The extent of platelet activation was determined qualitatively from the aggregates formation and quantitatively by the measurement of mean area surface of platelets with ImageJ 1.46r.

### 2.13. Statistical Analysis

GraphPad Prism v. 5.0 was used to perform the statistical analysis. Student's t-test or Mann-Whitney nonparametric test were used as appropriate. Results were expressed as mean±SD. P<0.05 was the criterion for statistical significance.

## 3. Results and Discussion

Novel techniques of stent surface modifications to resolve the problem of undesired clinical outcomes of intravascular implants are still an ongoing research area. Surface modification technologies can be divided into two main categories: passive and bioactive material surface and coatings [18]. Passive approaches are aimed at modification of surface chemistry or material physical structure [19]. Bioactive strategies employ either permanent immobilization of an active agent or local drug delivery [20].

Graphene, a single atom thick layer of sp2 carbon, has shown to be a suitable candidate for highly biocompatible, passive coating of the implant surface. Graphene is chemically inert and durable. Recently, the possible utilization of graphene for NiTi stents has been presented. NiTi stents, due to the various technical aspects of the deployment method, are routinely used in peripheral arterial disease but not for coronary interventions [21]. In this investigation we evaluated the potential of graphene coating on stainless steel 316L substrate for cardiovascular applications in coronary disease.

### 3.1. Spectroscopic Properties of Samples

**Figure 1:**
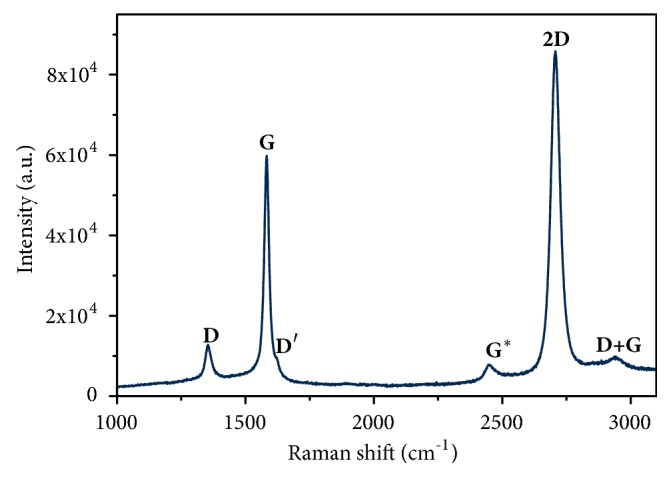
Raman spectrum of the graphene layer on stainless steel substrate (G-316-L).

To confirm the quality of obtained graphene layers after the transfer, Raman spectroscopy was carried out. The resulting spectrum (Figure 1) allows nondestructive identification of graphene layer by analyzing the spectral profile of G (1580 cm^−1^) and 2D (2690 cm^−1^) bands and the ratio between their intensities. The shape of the bands (sharp peaks) and the high 2D/G ratio clearly indicate the presence of a single or few layers of graphene [22].

Taking into account the difficulties of polymer-based transfer onto a nontypical substrate of mechanically polished steel, the achieved quality of the graphene layers confirmed by the Raman scattering measurement is exceptionally good. Due to the nature of the material, transferred from its pristine substrate onto another, the 2D/G ratio is smaller than 2. A value above 2 would be typical for a perfect graphene monolayer. A lower ratio is expected in materials obtained using such transfer methods. The other important bands in the interpretation of Raman spectra of graphene-related materials are the D (located at 1350 cm^−1^), D' (1620 cm^−1^), and D+G (2940 cm^−1^) [22]. In the evaluation of single- and few-layered graphene, all the bands mentioned above indicate defects in structure, with perfect monolayer being the standard model used for comparison. In an ideal case of single layer graphene sheet, those bands are completely absent or negligibly small. In particular, the presence of D band is associated with derogations from the perfect hexagonal crystalline structure. It is not observed in a system exhibiting ideal symmetry with no defects to be excited. The derogations include wrinkling, discontinuities and overlapping of the layers, all possible in the studied material as an effect of the nature of graphene transfer methods. All of the forementioned peaks are visible in the spectrum; however their intensity is very low in comparison to G and 2D peaks. The G*∗* peak at 2450 cm^−1^ is also visible although its interpretation in graphene and graphene-related structures is still a matter of debate. However, it is already known that this peak is absent in graphite spectra and was observed most frequently in single- and few-layered systems of graphene structures. It provides further indication that the obtained layers are of preferable properties.

### 3.2. AFM and SEM Characterization of Surfaces

**Figure 2:**
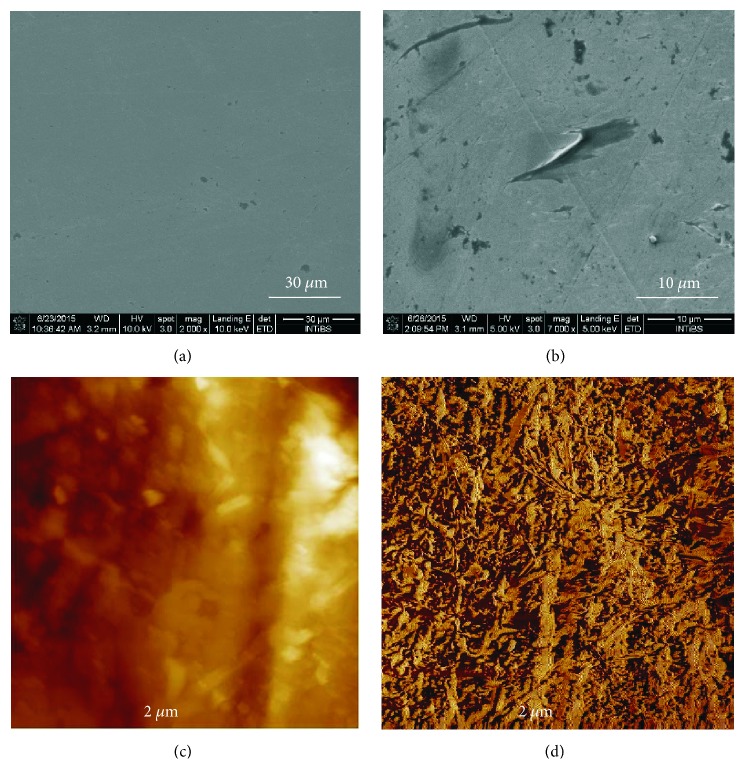
SEM images of the stainless steel surface: after mechanical polishing (a); after successful graphene transfer (b); AFM images of graphene coated steel (G-316-L): height image (c) and phase image (d). Figures 2(c) and 2(d) present AFM images of steel surface coated with graphene. The surface roughness was investigated and the surface profile in the selected cross-section and the average analyzed height of the film were determined.

SEM images of the obtained samples show that the layers are of good quality and their adhesion is exceptional. They remained on the substrate despite a more difficult target surface of the mechanically polished steel (in comparison to the perfectly flat surface of silicon wafers used in electronics) and intense chemical rinsing required for electron microscope imagery. The effects of the process are shown in Figure 2.

In our AFM study the relative height difference between the substrate and graphene sheets surface was (23.32 ± 5.65 nm). It is known that the height of graphene monolayer is about 0.76 nm [23]. The observed difference between the theoretical height of layer and the determined height of layer and a high value of standard deviation results most probably from the surface roughness caused by wrinkling and discontinuities of the obtained film. This suggests that the surface may be multifaceted or covered with ridged layer of graphene rather than creating uniform monolayer. Interestingly, the ridge-like surface feature may be even desired for the mechanically active structures coated with graphene, such as cardiovascular stents.

### 3.3. Low-Voltage SEM Surface Characterization

**Figure 3:**
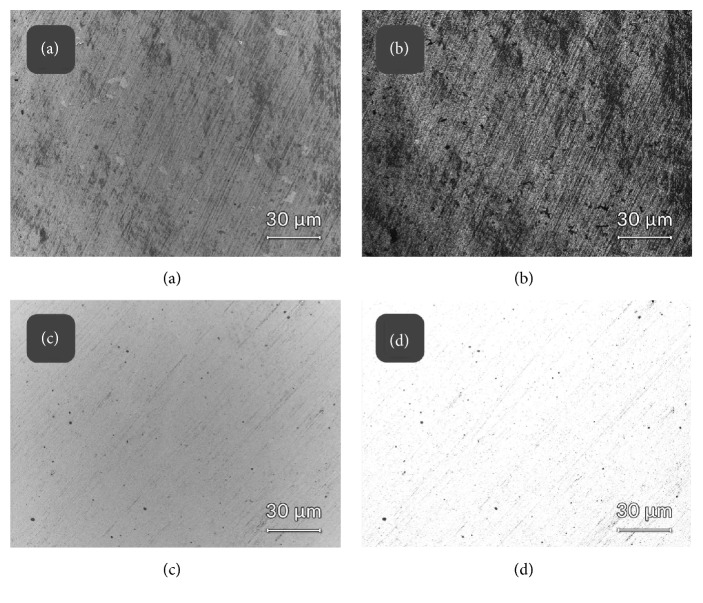
Low-voltage FE-SEM field-emission scanning electron microscopy with energy filtering. Graphene layers visualized ((a) and (b)) versus uncoated steel ((c) and (d)) discs. Detectors used: In-Lens SE1 detector ((a) and (c)); EsB energy-filtered backscattered electrons detector ((b) and (d)). Incident beam acceleration voltage 800 V used throughout the imaging, retarding potential of EsB detector grid set at 760 V (40 eV filtering energy difference).

EsB “in-Lens” detector allows blocking all electrons with kinetic energies lower than the strengths of the retarding field acting in front of the EsB detector [13]. As a result, it was possible to eliminate the secondary electrons from the image and include in the SEM detection the low energy-loss electrons only, aiming at identification of carbon contamination on steel [24]. It allowed to distinguish graphene coated discs from steel ones and to map the graphene distribution over the substratum. Images shown in Figure 3 represent electron EsB energy-filtered maps, correlated with the In-Lens detection directly from the sample surfaces, without coating or any additional contrasting applied, to approach the native composition of biosample and original substrate composition as close as possible. Material contrast was generated by energy filtering approach (EsB detector, panels band, (d)) and the images were recorded at identical settings; thus they are directly comparable. Please note darker appearance of graphene when compared to noncoated steel ((b) versus (d), respectively).

### 3.4. Endothelial Cell Adhesion and Proliferation

**Figure 4:**
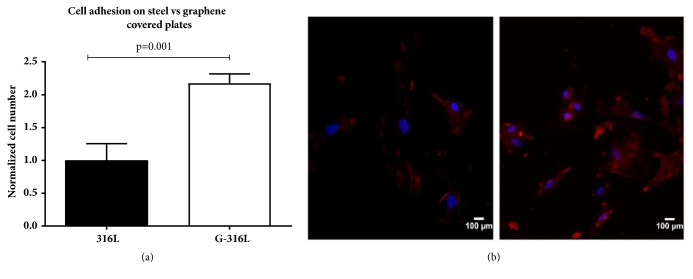
An adhesion of endothelial cells on graphene coated discs (G-316L). Cell number on G-316L was normalized to cell number on still substrate (316L) which served as a control (a). Fluorescent stained endothelial cells on steel (left) and graphene discs (right) (b); blue-nuclei; red-cytoplasm; n=6 per group, mean±SD.

To evaluate whether steel discs coated with a single layer of graphene enhance cell adhesion to the surface, steel discs and graphene coated discs were covered with 1 x 10^4^ of endothelial cells per cm^2^ and incubated in conditions described in* Materials and Methods*. After 24h of cultivation, the cell nuclei were stained with DAPI and cell bodies were stained with antiendothelium conjugate to facilitate visualization and counting. Results showed that G-316L surface enhanced the adhesion of endothelial cells 24 h after seeding in comparison to uncoated 316L steel (Figures 4(a) and 4(b)).

**Figure 5:**
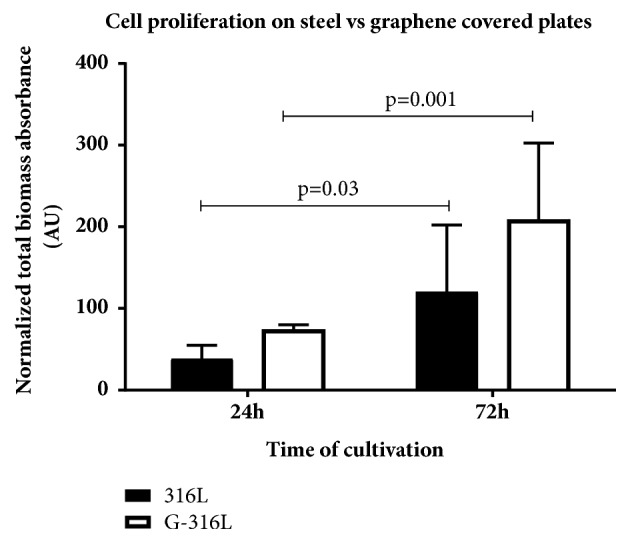
The comparison of cells proliferation on steel (316L) and graphene (G-316L) discs. Cell proliferation was assessed by the measurement of total biomass absorbance after 24 and 72 hours of cultivation; n=6 per group, mean±SD.

316L and G-316L discs were covered with endocardial endothelial cells (1 x 10^4^ cells per cm^2^) to evaluate whether graphene creates more favorable conditions for cell proliferation than steel does. To assess the rate of proliferation, an increase of biomass (by means of sulforhodamine staining) between 24h and 72h of cultivation was determined. The data showed more enhanced proliferation of endothelium on graphene-overlaid discs (3 times greater number of cells after 72h in comparison to number of cells adhering at 24h of cultivation) than on steel discs (2.4-fold increase in the same time period), p=0.001 (Figure 5).

It was documented that graphene is able to change the cell transport properties and strongly interact with the cell by large surface area [25]. Endothelial cells are anchorage-dependent cells which need to initially adhere to substrates in order to spread, proliferate, and perform their functions. All the above effects of graphene covered steel substrate on the function of HECs were determined in this investigation. In this study the HEC attachment to the bare 316L stainless steel and graphene covered 316L was evaluated after 24h of aerobic cultivation. As presented on Figure 4 the number of cells adhering to graphene was twice as large as the number of cells adhering to steel. The effect reached statistical significance (p =0.001). Moreover, the total biomass indicating cell proliferation rate after next 48h increased over 100% on graphene, while cells on steel discs did not proliferate so effectively.

This confirms that graphene covered 316L substrate is highly biocompatible for endothelial cell adhesion and proliferation and these are the desired effect of stent placement (Figure 5).

### 3.5. Vitality and Metabolic Activity of Endothelial Cells on Graphene Covered 316-L Discs (G-316L)

Main influencing factors for cytotoxicity of nanomaterials are their chemical composition, size, and shape. It is well known that also a kind of carbonaceous nanomaterials plays an important role in interactions with cells, tissues, and organism [26]. Therefore, the possibility of cytotoxic effect of transferred graphene layers on human endothelial cell line has been also tested.

**Figure 6:**
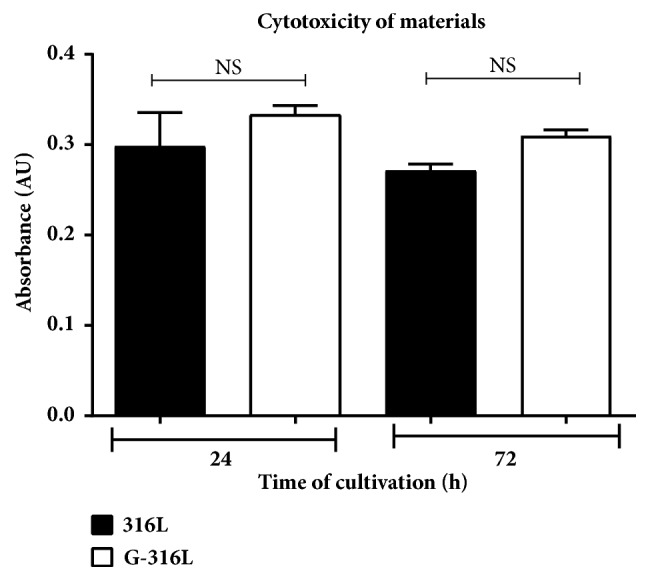
The assessment of cytotoxic effect of graphene (G-316L) on endothelial cells in comparison to steel (316L) during 24 and 72 hours of cultivation; n=6 per group, mean±SD.

To evaluate whether graphene coated discs and steel discs are cytotoxic to endothelial cells, an* In Vitro* Toxicology Assay Kit (Sigma Aldrich, USA) has been used and cell vitality was assessed by trypan blue staining. The experiment showed no cytotoxic effect on either steel or graphene-overlaid discs 24h and 72h after cultivation (Figure 6). This was confirmed by living cells' number obtained by trypan blue staining. Viability of the cells cultivated on steel and graphene-overlaid discs was over 95% in both cases.

We have also tested the metabolic activity of endothelium on 316L material. It is well known that biomaterial may affected cells adherence and proliferation as well as their function. Endothelial cells are anchorage-dependent cells which need to initially adhere to substrate in order to spread and proliferate and then to perform their function. We tested if, apart from their adhesion and proliferation, their metabolic function is maintained.

**Figure 7:**
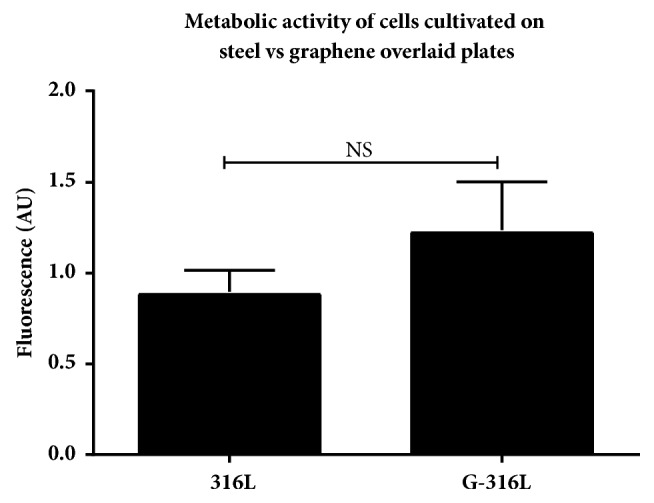
Metabolic activity of endothelial cells cultivated on graphene (G-316L) and steel (316L) discs; n=6 per group, mean±SD.

Metabolic activity of cells cultivated for 72h on 316L discs and G-316L discs was assessed by FDA test. FDA-fluorescein diacetate, cell-permeant esterase substrate, served as a substrate for the measurement of enzymatic activity of the cells. The data showed that endothelial cells cultivated on G-316-L surface exhibited greater metabolic activity than cells cultivated on 316L alone, although the difference was not statistically significant (Figure 7). As the first we showed that graphene coated 316L potential stents allow for maintaining of proper metabolic function of endothelial cells.

Similarly, to study of Podila et al. involving smooth muscle cells (SMCs) and Rat Aortic Endothelial Cells (RAECs) [21], we showed that coating with chemically inert and atomically smooth graphene does not affect the viability and metabolism of human primary coronary artery endothelial cells (HPCAEC) after 72 hours of incubation compared to uncoated 316L substrate (Figures 6 and 7). This is a very important observation in a view of implementation of graphene coatings as the surface biomaterial for intracoronary stents in humans. Nonetheless, the potential long-term adverse effects of graphene functionalized implants cannot be excluded, and this issue requires further studies* in vivo*.

### 3.6. Assessment of Endothelial-to-Mesenchymal Transition (EndoMT) on Graphene Covered 316L

**Figure 8:**
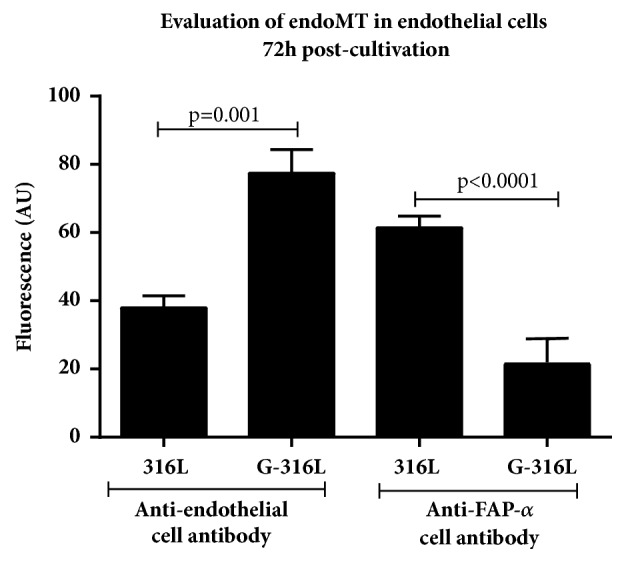
Endothelial-to-mesenchymal transition on graphene (G-316L) coated in comparison to steel (316L) discs; n=6 per group, mean±SD.

To evaluate whether G-316L surface has a potential to influence the endothelial cells phenotype, endothelial-to-mesenchymal transition (EndoMT) has been determined. The endothelium cultivated on uncoated steel and graphene coated discs was stained with conjugate containing anti-human FAPa (fibroblast activating protein alpha) antibody (indicating fibrocytes phenotype) or anti-human endothelium antibody (indicating endothelium phenotype) and DyLight 488 or DyLight 550 secondary antibody, respectively. Interestingly, the experiment showed greater number of fibroblast phenotype cells on uncoated steel discs and greater number of endothelium phenotype cells on graphene-overlaid discs (p<0.05) (Figure 8).

Kato et al. showed that surgical and catheter-based interventions on pulmonary veins were associated with pulmonary vein stenosis due to endothelial-mesenchymal transition. The loss of endothelial and gain of mesenchymal marker expression or coexpression of endothelial and mesenchymal antigens in banded pulmonary vein intimal cells was observed [27]. EndoMT is closely regulated by a wide range of signaling pathways and transcription factors, including vascular endothelial growth factor (VEGF), NOTCH, and Snail and Slug (VEcadherin, *α*-SMA and fibronectin expression) [28]. Since graphene surface layer obtained in this study was characterized as nontoxic and biocompatible material, we investigated for the first time whether graphene surface can also influence the undesired endothelial-to-mesenchymal transition. We showed that the fluorescence of anti-endothelial cell antibody was approximately twice as large on graphene covered steel substrates than on steel with statistical significance (p≤0.001). At the same time there was three times less fluorescence of anti-FAP alpha cell antibody on graphene than on steel (p<0,001) (Figures 6 and 8). Our study showed that G-316L discs also enhanced the growth of endothelial cells; however this is the pioneer study showing that graphene coating stainless steel discs influence the undesired transition of endothelial-to-mesenchymal cells phenotype. Since increased proliferation of smooth muscle cells as well as endothelial-to-mesenchymal transition due to inflammatory response is main cause of restenosis, diminished promesenchymal cells transduction and hence neointima formation due to graphene coating of potential stainless steel stents are a unique and promising feature.

### 3.7. Platelet Adhesion Tests

**Figure 9:**
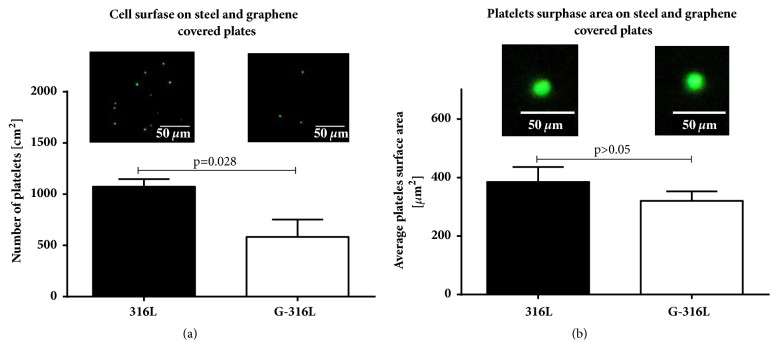
Platelets attachment (adhesion) (a) and mean platelets surface area (b) on steel (316L) and graphene (G-316L) coated discs. A representative fluorescence images of platelets are shown in upper part of the figures; n=6 per group, mean±SD.

To determine whether graphene covered discs adversely affect the platelets adhesion and/or activation, G-316L and 316L discs were covered with a large number of platelets according to protocol described earlier. The data showed that the number of platelets attached to G-316L surface was approximately two times lower than to uncoated steel (p=0,028) (Figure 9(a)). Moreover, we observed that mean surface area of platelets adhering to G-316L was smaller than the one on 316L (Figure 9(b)).

Blood platelets adhesion to the biomaterials surface is one of the primary indicators of their hemocompatibility [29]. The platelets adhesion to both 316L substrate and graphene covered 316L substrate was measured to preliminarily evaluate the blood compatibility. The number of platelets adhering to graphene surface was approximately two times lower than to steel surface (p=0.028). The surface area of the platelets adhering to graphene surface was also lower than those adhering to steel surface as shown on Figure 7. Reduced platelet adhesion to graphene surfaces can be imputed to inert properties of graphene coating and may potentially decrease undesired consequences of stent implementation. The precise mechanism of G-316L hemocompatibility needs further investigation.

## 4. Conclusions

The high-quality CVD-grown graphene has been moved to the steel (316L) substrate using the PMMA-based method. The achieved quality of the transferred graphene layer was exceptionally good and confirmed by the Raman scattering measurement, low-voltage FE-SEM images, and the AFM analysis. It was confirmed that graphene coating on 316L (G-316L) supports the adhesion and proliferation of human primary coronary artery endothelial cells (HPCAEC) to a greater extent than the uncoated substrate. It was proved also that obtained surface has a unique potential to affect the endothelial cell phenotype by diminishing the endothelial-to-mesenchymal transition (EndoMT) and thus stronger reducing the risk of in-stent restenosis. This finding further confirms the biocompatibility of graphene coating. All of these characteristics of the coating along with excellent mechanical properties of 316L make a G-316L a suitable candidate for intravascular implants.

## Data Availability

All data used to support the findings of this study are included within the article.

## Supplementary Materials

Supplementary Materials Schematic presentation of endothelial cells and blood platelets interactions with the graphene coated cardiovascular stent surface.
