# Supplementary figures and images for: Biocompatible Carbon-Based Coating as Potential Endovascular Material for Stent Surface
Source: Biomed Res Int. 2018 Oct 4;2018:2758347. doi: 10.1155/2018/2758347 (PMC6193326; doi:10.1155/2018/2758347)

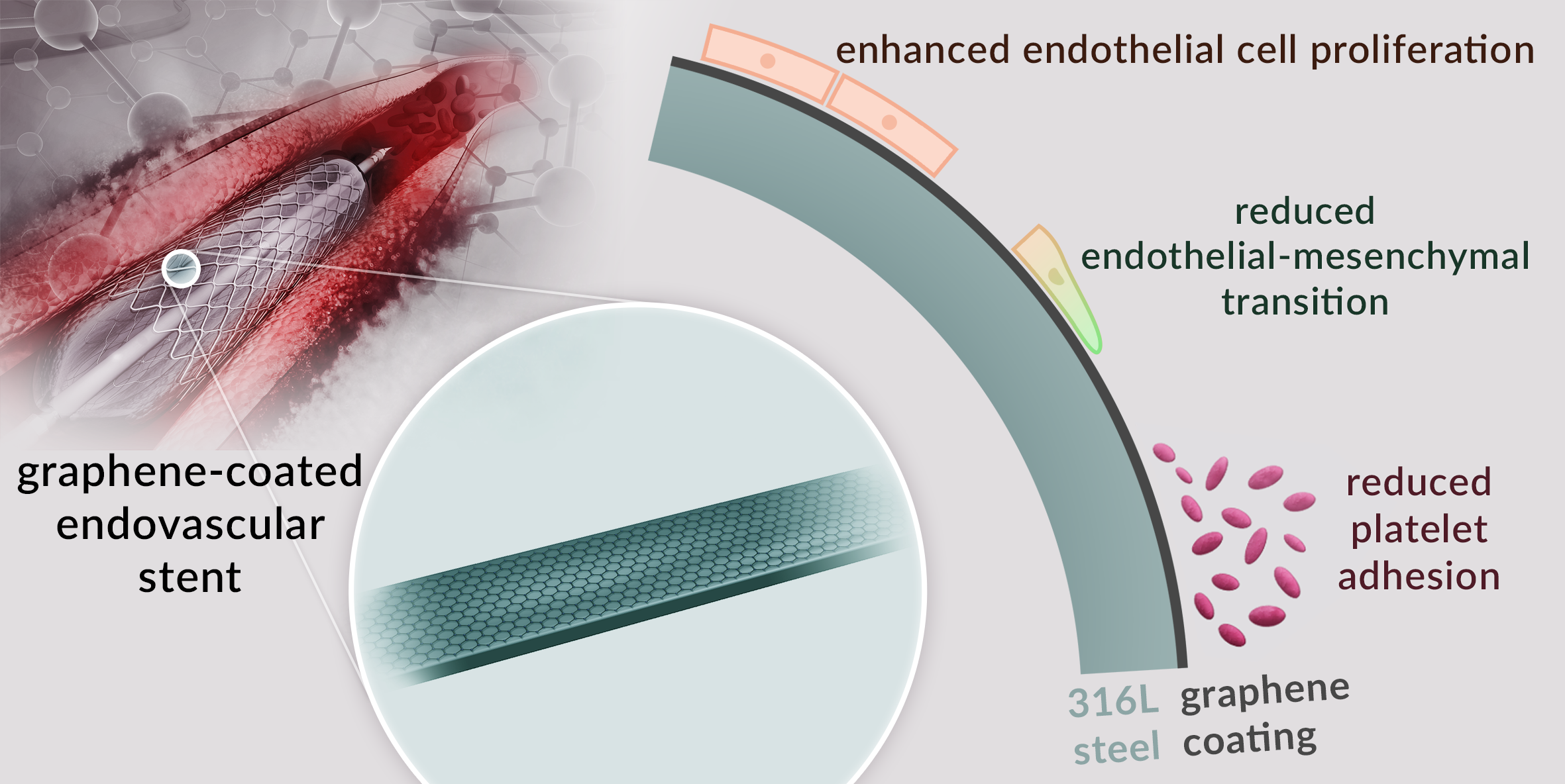

Supplement: Supplementary Materials — Schematic presentation of endothelial cells and blood platelets interactions with the graphene coated cardiovascular stent surface. [file 2758347.f1.tif]
